# Spatial heterogeneity and scenario simulation of carbon budget on provincial scale in China

**DOI:** 10.1186/s13021-023-00237-x

**Published:** 2023-09-20

**Authors:** Zhenyue Liu, Jinbing Zhang, Pengyan Zhang, Ling Jiang, Dan Yang, Tianqi Rong

**Affiliations:** 1 College of Geography and Environmental Science, Henan University, Kaifeng, 475004 Henan China; 2 School of Urban Economics and Public Administration, Capital University of Economics and Business, Beijing, 100070 China; 3 Xinyang Vocational and Technical College, Xinyang, 464000 Henan China; 4 School of Government, Central University of Finance and Economics, Beijing, 100081 China

**Keywords:** Carbon budget, Spatial autocorrelation, Decoupling, Scenario simulation, China

## Abstract

**Background:**

Conducting an extensive study on the spatial heterogeneity of the overall carbon budget and its influencing factors and the decoupling status of carbon emissions from economic development, by undertaking simulation projections under different carbon emission scenarios is crucial for China to achieve its targets to peak carbon emissions by 2030 and to achieve carbon neutrality by 2060. There are large disparities in carbon emissions from energy consumption, the extent of land used for carbon absorption, and the status of decoupling of emissions from economic development, among various regions of China.

**Results:**

Based on night light data and land use data, we investigated carbon budget through model estimation, decoupling analysis, and scenario simulation. The results show that the carbon deficit had a continuous upward trend from 2000 to 2018, and there was a significant positive spatial correlation. The overall status of decoupling first improved and then deteriorated. Altogether, energy consumption intensity, population density of built-up land, and built-up land area influenced the decoupling of carbon emissions from economic development. There are significant scenarios of carbon emissions from energy consumption for the study area during the forecast period, only in the low-carbon scenario will the study area reach the expected carbon emissions peak ahead of schedule in 2027; the peak carbon emissions will be 6479.27 million tons.

**Conclusions:**

China’s provincial-scale carbon emissions show a positive correlation with economic development within the study period. It is necessary to optimize the economic structure, transforming the economic development mode, and formulating policies to control the expansion of built-up land. Efforts must be made to improve technology and promote industrial restructuring, to effectively reduce energy consumption intensity.

## Background

Climate issues marked by global warming, are a major problem faced by mankind in the twenty-first century [1], which has a significant negative impact on the economic, social, and ecological environment worldwide [2, 3]. The key to slowing down the global warming trend is to reduce CO_2_ emissions, which has currently become the focus of global attention and consensus [4]. Since the opening up of China following economic reforms and rapid economic development, China’s energy consumption has continued to rise, and carbon emissions have increased. China has also become the world’s largest carbon emitter [5], and is actively participating in global climate governance. In 2015, China proposed a National Independent Contribution Plan, promising to reduce CO_2_ emissions per unit of GDP by 60–65% by 2030 compared to 2005 [6]. In 2020, during the 75th United Nations General Assembly, China also proposed to strive to achieve the “dual carbon” targets of peaking CO_2_ emissions by 2030 and achieving carbon neutrality by 2060 [7].

The carbon budget is important for achieving the “dual carbon” targets, and has been an area of interest for scholars [8, 9]. The carbon budget mentioned in this paper refers to a collective term that includes both carbon emissions and carbon absorption. The study of the carbon budget is essentially the study of carbon sources and sinks; carbon emissions and carbon absorption correspond to carbon sources and carbon sinks, respectively. Researchers have mainly focused on the carbon balance at different scales and of different land use types. In terms of the spatial heterogeneity of carbon budgets at different scales, scholars have conducted many studies at global [10, 11], intercontinental [12, 13], and national [14] scales. Chinese scholars have mainly conducted studies at the national [15], provincial [16], and municipal [17] levels. As for the carbon budget of different land use types, many studies have been conducted on individual land use types such as forestland [18, 19], grassland [20], water land [21], and built-up land [22]. Based on the study of the carbon budget, researchers have conducted analysis of the influencing factors, with the decomposition of influencing factors mainly focused on carbon emissions. Among the decomposition methods for influencing factors, index decomposition [23], structural decomposition [24], regression analysis [25], gray correlation analysis [26], and Data envelopment analysis (DEA) model [27] are the most frequently used. The logarithmic mean divisia index (LMDI) decomposition method does not require input-output data, compared with the structural decomposition method, and does not require input-output coefficients compared with the Laspeyres index decomposition method [28]. The LMDI decomposition method has been used to decompose the drivers of overall regional carbon emissions [29, 30], industrial carbon emissions [31], and carbon emissions from household consumption [32], and substantial research results have been obtained. The relationship between carbon emissions and economic development has become an area of research interest [33]. A widely used method is decoupling analysis, where the status of decoupling of carbon emissions from economic development, at various scales such as global [34], national [35], and provincial [36], and in different industries such as construction [37], industry [38, 39], and tourism [40] are investigated. We combined decoupling analysis with the LMDI decomposition method to quantitatively analyze the effectiveness of the contribution of each influencing factor to the decoupling of carbon emissions from economic development. Based on the analysis of the contribution of the influencing factors to carbon emissions, some scholars forecast the future evolution of carbon emissions in the study area through scenario setting, and predicted the peak or structural evolution of carbon emissions in the construction sector [41], road passenger transportation sector [42], and the power sector [43]. The above studies have analyzed the spatial heterogeneity of the carbon budget of energy consumption and its influencing factors at different scales using various methods, and have yielded substantial results. However, most of the studies have analyzed the carbon emissions of individual industries or the carbon absorption of a single land use type, and relatively few studies have analyzed the spatial heterogeneity of the overall carbon budget of energy consumption in China and provided the projected carbon emissions for future dates.

**Fig. 1:**
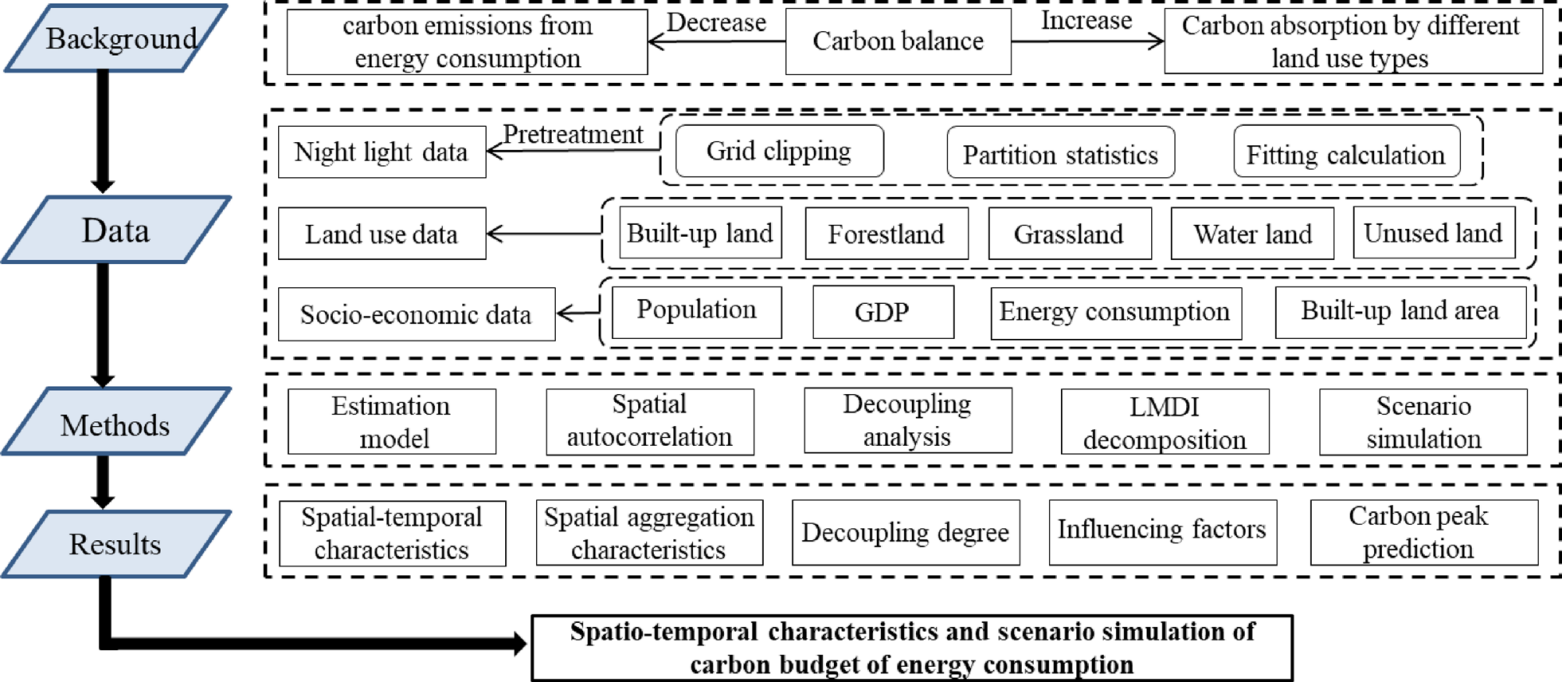
Research framework diagram

China is a vast country with large differences in resource endowment, population, socio-economic development levels, industrial infrastructure, and land use type composition, resulting in variability in carbon emissions, and carbon absorption from land use, for different regions [44]. The carbon emission reduction targets defined at the national macro level need to be implemented at the regional level. Therefore, it is necessary to estimate the carbon budget of each province in China, and further analyze the spatial heterogeneity and factors influencing carbon emissions in the study area. Scenario analysis based on the contribution of the influencing factors to the increase in carbon emissions must be performed, so as to predict the future trend of carbon emissions from energy consumption in the study area and forecast the year when carbon emissions would peak (Fig. 1). By exploring the degree of influence of each contributing factor on the carbon budget, and the future trend of carbon emissions, we want to explore the following questions: (1) How has the decoupling of carbon emissions and economic development in various provinces of China evolved? (2) Can China achieve its goal of peaking carbon emissions by 2030? We aim to provide a reference for the formulation of reasonable and targeted regional emission reduction policies, and also provide a reference for other developing countries to explore low-carbon development paths.

## Methods

### Study area

The study area includes 30 provincial administrative regions of China, including 22 provinces, four municipalities directly under the central government, and four autonomous regions. Tibet, Hong Kong, Macao, and Taiwan were not included, considering the unavailability of relevant data.

### Carbon emission estimation model

Night light data and data on carbon emissions were used to build a carbon emission estimation model. The hypothesis is that there is a correlation between the digital number (DN) values and carbon emissions; that is, higher the DN value, higher the carbon emissions, and there is a consistent correlation between the DN values at the provincial scale and at the grid scale [45]. Curvilinear simulations of both data were conducted using SPSS 25 software (International Business Machines Corporation). We found that there was a good linear correlation between the DN value of night light data and carbon emissions. An estimation model was constructed, with the sum of the night light grid values at the provincial scale, and the corresponding provincial energy consumption carbon emissions as its components. The model structure is as follows:

1 $$\ln {C_{E}} = 0.64 \ln NTL + 1.516$$

where *C*_*E*_ is the carbon emissions from energy consumption, and *NTL* is the night light data grid value; the goodness-of-fit of the model reaches 0.694.

### Carbon absorption estimation model

In studies related to carbon emissions from land use, built-up land and cultivated land are carbon sources, while forestland, grassland, water land, and unused land are carbon sinks which therefore need to be considered in calculation of carbon absorption:

2 $$C_{S} \, = \,\sum\limits_{{i = 1}}^{4} {A_{i} } \, \times \,a_{i}$$

where *C*_*S*_ denotes the total carbon absorption, *A*_*i*_ denotes the area of land use type *i*, and *a*_*i*_ denotes the carbon absorption coefficient for land use type *i*. The carbon absorption coefficients of each land use type were derived from previous studies (Table 1) [46, 47].

**Table 1:** Carbon absorption coefficient of different land use types

Land use	Forestland	Grassland	Water land	Unused land
Coefficient/(t C/hm^2^/a)	0.644	0.022	0.253	0.005

### Carbon deficit estimation model

3 $$C_{D} \, = \,C_{E} \, - \,C_{S}$$

where *C*_*D*_ denotes carbon deficit or surplus, *C*_*E*_ denotes regional carbon emissions, and *C*_*S*_ denotes regional carbon absorption. When *C*_*D*_ > 0, carbon emission is greater than carbon absorption, indicating that the region is a carbon source and has carbon deficit; when *C*_*D*_ = 0, the region is in carbon balance; when *C*_*D*_ < 0, carbon emission is less than carbon absorption, indicating that the region is a carbon sink and its carbon balance is positive.

### Spatial autocorrelation

Moran’s index or Moran’s *I* was proposed in 1950 to test whether a phenomenon is spatially clustered, and to describe the spatial characteristics of its distribution in the study area [48]. Spatial autocorrelation analysis methods include global and local spatial autocorrelations. The global Moran’s *I* for the study area was calculated as follows:

4 $$I\, = \,\frac{{k\sum\limits_{{p = 1}}^{m} {\sum\limits_{{q = 1}}^{m} {W_{{pq}} \,\left( {x_{p} \, - \,\bar{x}} \right)\left( {x_{q} \, - \,\bar{x}} \right)} } }}{{\left( {\sum\limits_{{p = 1}}^{m} {\sum\limits_{{q = 1}}^{m} {W_{{pq}} } } } \right)\sum\limits_{{p = 1}}^{m} {\left( {x_{p} \, - \,\bar{x}} \right)^{2} } }}$$

where *x*_*p*_ denotes carbon deficit in area *p*, *x*_*q*_ denotes carbon deficit in area *q*, ${\bar{x}}$ denotes the average carbon deficit in the study area, and *m* denotes the number of areas, * W*_*pq*_ is the spatial weight matrix. The inverse of the quadratic distance of geographic units was chosen as the spatial weight matrix in this study. The closer *I* is to 1, the more significant is the positive spatial correlation, and the closer it is to -1, the more significant is the negative spatial correlation. The global Moran’s *I* can only evaluate overall distribution and trend, and cannot determine the spatial correlation of independent units. Spatial heterogeneity exists at the level of spatial autocorrelation, that is, the degree of spatial correlation varies from one local area to another. Therefore, local spatial autocorrelation is used to describe local spatial heterogeneity and to identify the spatial distribution patterns of carbon deficit. The local Moran’s *I*, Moran’s *I*_*c*_, was calculated using the following formula:

5 $$I_{c} \, = \,\frac{{m\,\left( {x_{p} \, - \,\bar{x}} \right)}}{{\sum\limits_{{p = 1}}^{m} {\left( {x_{p} \, - \,\bar{x}} \right)^{2} } }}\sum\limits_{{p \ne q}}^{m} {W_{{pq}} \,\left( {x_{q} \, - \,\bar{x}} \right)}$$

Each variable in Eq. (5) represents the same parameter as in the formula for global Moran’s *I*. Local agglomeration patterns are further distinguished by Moran’s *I*_*c*_, and include H-H agglomeration (high-observed area surrounded by high-observed areas), L-L agglomeration (low-observed area surrounded by low-observed areas), L-H agglomeration (low-observed area surrounded by high-observed areas), and H-L agglomeration (high-observed area surrounded by low-observed areas).

### Decomposition of influencing factors

The degree of decoupling is an important indicator of the coupling between economic development and environmental pressures in a region, and reflects the sensitivity of changes in resource and environmental pressures to economic changes [49]. A decoupling model is constructed whose structure is as follows:

6 $$D\, = \,\frac{{\Delta C_{D} /C_{{D0}} }}{{\Delta GDP/GDP_{0} }}\, = \,\Delta C_{D} \, \times \,\left( {\frac{{GDP_{0} }}{{\Delta GDP\, \times \,C_{{D0}} }}} \right)$$

where *D* is the degree of decoupling (refer to Table 2 for specific criteria) [28], $\varDelta {C}_{D}$ and $\varDelta GDP$ are the changes in the regional carbon deficit and GDP at the end of the base period, respectively. *C*_*D*0_ and *GDP*_*0*_ are the carbon emissions from energy consumption and GDP during the base period, respectively.

**Table 2:** Classification of decoupling status

Degree of decoupling		*D*	$\varDelta C$	$\varDelta GDP$
Negative decoupling	Expansive negative decoupling	*D* ≥ 1.20	> 0	> 0
	Weak negative decoupling	0 ≤ *D* < 0.80	< 0	< 0
	Strong negative decoupling	*D* < 0	> 0	< 0
Decoupling	Recessive decoupling	*D* ≥ 1.20	< 0	< 0
	Weak decoupling	0 ≤ *D* < 0.80	> 0	> 0
	Strong decoupling	*D* < 0	< 0	> 0
Coupling	Expansive coupling	0.80 ≤ *D* < 1.20	> 0	> 0
	Recessive coupling	0.80 ≤ *D* < 1.20	< 0	< 0

The LMDI decomposition method was proposed in the 1990s [50], and has the advantages of mature technology, variety in forms, easy calculation, and no residuals in the decomposition [51]. The LMDI decomposition method can decompose the factors into the carbon emission coefficient, energy intensity, GDP per capita, population density of built-up land, and built-up land area.

7 $$C\, = \,\frac{C}{E}\, \times \,\frac{E}{{GDP}}\, \times \,\frac{{GDP}}{P}\, \times \,\frac{P}{A}\, \times \,A$$

8 $$\Delta C\, = \,\Delta CE\, + \,\Delta EG\, + \,\Delta GP\, + \,\Delta PA\, + \,\Delta A$$

9 $$\begin{array}{ll}{\begin{aligned}\Delta CE=\frac{{C_T} - {C_0}}{\ln {C_T} - \ln {C_0}} \times \ln \frac{C{E_T}}{C{E_0}} \hfill \\ \Delta EG=\frac{{C_T} - {C_0}}{\ln {C_T} - \ln {C_0}} \times \ln \frac{E{G_T}}{E{G_0}} \hfill \\ \Delta GP=\frac{{C_T} - {C_0}}{\ln {C_T} - \ln {C_0}} \times \ln \frac{G{P_T}}{G{P_0}} \hfill \\ \Delta PA=\frac{{C_T} - {C_0}}{\ln {C_T} - \ln {C_0}} \times \ln \frac{P{A_T}}{P{A_0}} \hfill \\ \end{aligned}} & \Delta A\, = \,\frac{C_{T} \, - \,C_{0} }{\ln C_{T} \, - \,\ln C_{0} }\, \times \,\ln \frac{A_{T} }{A_{0}}\\ \end{array}$$

where *E* is the energy consumption, *P* is the population, and *A* is the built-up land area. $\varDelta CE$, $\varDelta EG$, $\varDelta GP$, $\varDelta PA$, and $\varDelta A$are the changes in the contributions of carbon emission factor, energy intensity, GDP per capita, population density of built-up land, and built-up land area, respectively. $\varDelta CE$ should be 0, but the carbon emission data used in this study were obtained by fitting the night light data with the energy consumption data due to which there is a certain error; the actual amount of carbon deficit is hence applied, resulting in $\varDelta CE$ in this study not being 0. Therefore, when calculating the contribution of each influencing factor to decoupling, the contribution to carbon deficit generated by the carbon emission coefficient was eliminated first. In addition, as the strong influence of economic output on carbon reduction suppresses the influence of other factors, the contribution of GDP per capita was also excluded.

10 $$\Delta F\, = \,\Delta C\, - \,\Delta CE\, - \,\Delta GP\, = \,\Delta EG\, + \,\Delta PA\, + \,\Delta A$$

11 $$Z\, = \, - \,\frac{{\Delta EG\, + \,\Delta PA\, + \,\Delta A}}{{\Delta GP}}\, = \,Z_{{EG}} \, + \,Z_{{PA}} \, + \,Z_{A}$$

where *Z* represents the effectiveness of the contribution of each influencing factor to decoupling, *Z*_*EG*_, *Z*_*PA*_, and *Z*_*A*_ being the corresponding contributions of energy intensity, GDP per capita, built-up land population density, and built-up land area, respectively. When *Z* ≥ 1, it means that the factor contributes strongly to decoupling; when 0 < *Z* < 1, the contribution of the factor to decoupling is weak, and when *Z* ≤ 0, the factor does not contribute to decoupling or hinder decoupling [28].

### Scenario simulation

Since carbon absorption is mainly influenced by the composition and extent of land subject to different types of use, not by factors such as population, economy and energy consumption, the development scenario was set to explore the trend in carbon emissions only. Taking 2030 (the year carbon emissions are expected to peak) as the year in focus, based on possible future trends in changes in the contributions of the four indicators to carbon emissions in the study area, and referencing existing research [52, 53], this study set up three scenarios: high-carbon, basic, and low-carbon scenarios. The future annual change in carbon emissions was predicted by combining the contributions of the influencing factors using the following equation:

12 $$\Delta C\, = \,\Delta EG\, \times \,(1\, + \,n_{1} )\, + \,\Delta GP\, \times \,(1\, + \,n_{2} )\, + \,\Delta PA\, \times \,(1\, + \,n_{3} )\, + \,\Delta A\, \times \,(1\, + \,n_{4} )$$

where $\varDelta C$ denotes the change in carbon emissions from energy consumption between adjacent years;$\varDelta EG$, $\varDelta GP$, $\varDelta PA$, and $\varDelta A$ respectively denote the contributions of energy consumption intensity, GDP per capita, population density of built-up land, and built-up land area, to carbon emissions in the previous year, respectively. *n*_*1*_, *n*_*2*_, *n*_*3*_, and *n*_*4*_ denote the annual average rates of change in the contribution of these four influencing factors, respectively. $\varDelta C$ > 0 indicates an increase in carbon emissions; $\varDelta C$ = 0 indicates that carbon emissions remain unchanged; $\varDelta C$ < 0 signifies a reduction in carbon emissions. Adopting 2000–2018, 2005–2018, 2010–2018, and 2015–2018 as the reference periods for setting the parameters of the study scenarios, the median of the annual average values for the rate of change in the contribution of each factor in the four stages is set as the indicator of the rate of change for the basic scenario. There are two median values of annual average change rate of factor contribution in the four periods, considering the degree of differential impact of different periods on the future, only one median value of annual average change rate of factor contribution for the period closer to the present was maintained [54]. The high-carbon and low-carbon scenarios are set at certain intervals based on the basic scenario [55], with the indicator values under the low-carbon scenario set with reference to the relevant planning targets proposed by China.

High-carbon scenario: This scenario is representative of the situation where China’s main objective is economic development, adopting a careless approach that pays less attention to pollution, carbon emissions, and climate change. In this scenario, rapid economic development will inevitably lead to high energy consumption and environmental pollution; the impact of built-up land area and GDP per capita on the increase in carbon emissions will be enhanced, while the impact of energy consumption intensity and population density of built-up land will be weakened.

Basic scenario: Based on the overall carbon emission evolution trend for the study area from 2000 to 2018, this scenario reflects the future carbon emission trend for each provincial administrative region according to its energy intensity status, GDP per capita, population density of built-up land, and built-up land area in the study period, in the absence of any government policy intervention.

Low-carbon scenario: This scenario is representative of a situation where China no longer focuses on economic development as its main goal, but on an energy-saving and low-carbon development approach. Each provincial administrative region will fully consider the future social, economic, and environmental development needs, and implement various measures such as energy conservation, emission reduction, and industrial structure optimization to achieve sustainable and low-carbon development. In the context of China’s pursuit of the “dual carbon” target, this scenario is more in line with the future development trend.

## Results

### Spatial heterogeneity of carbon budget

**Fig. 2:**
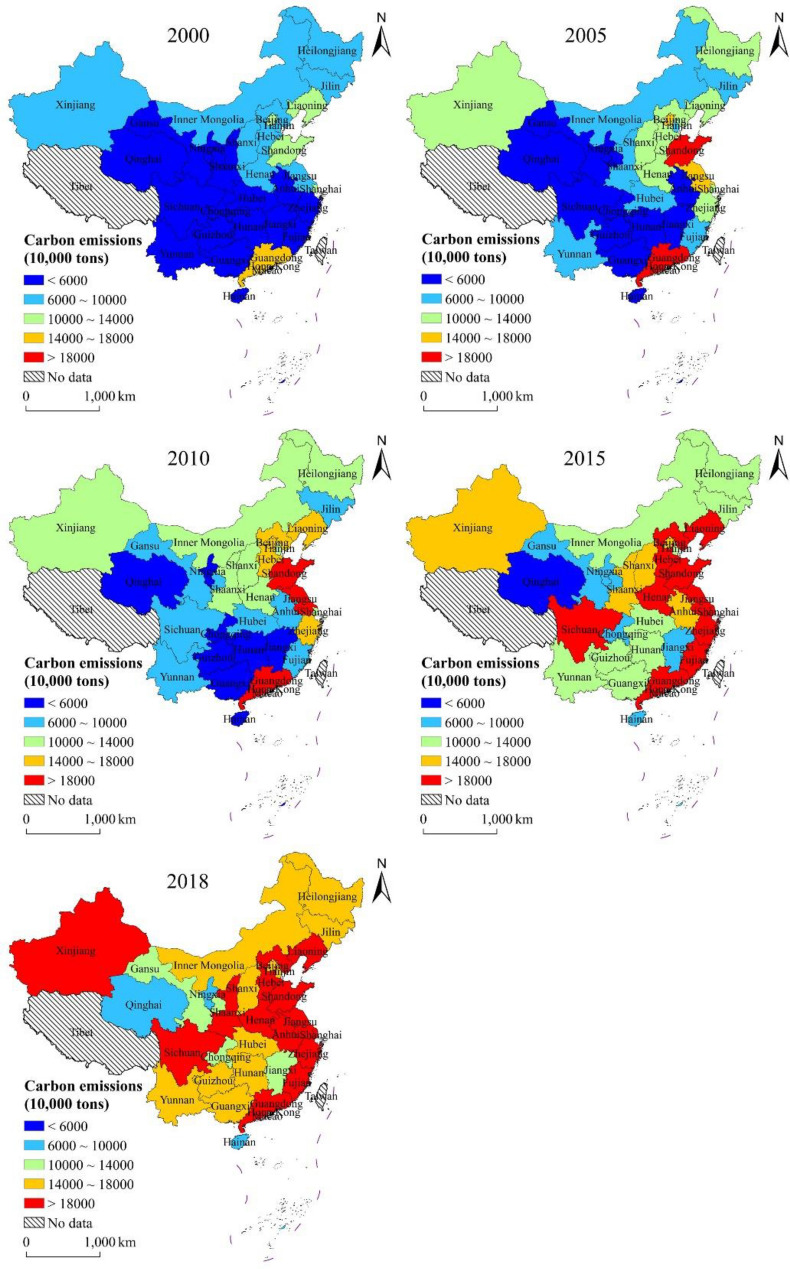
Spatial distribution of carbon emissions from energy consumption at provincial level

**Fig. 3:**
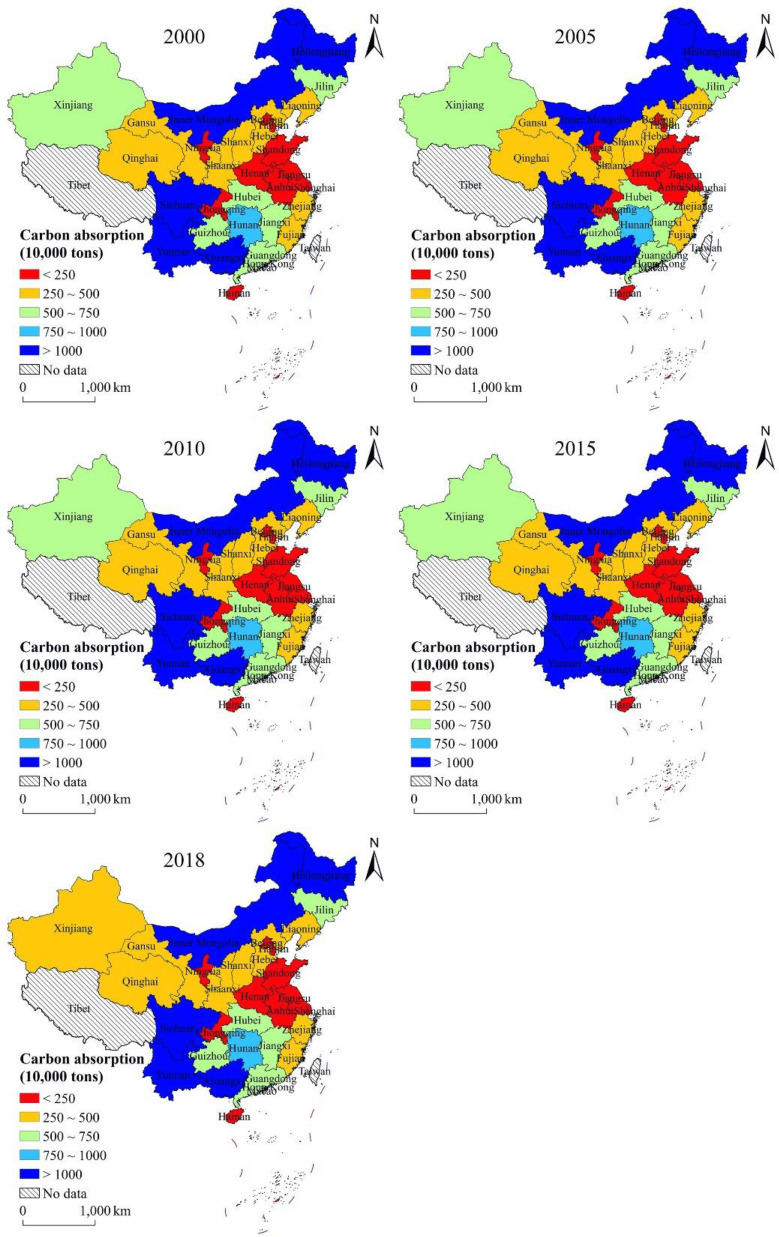
Spatial distribution of carbon absorption at provincial level

**Fig. 4:**
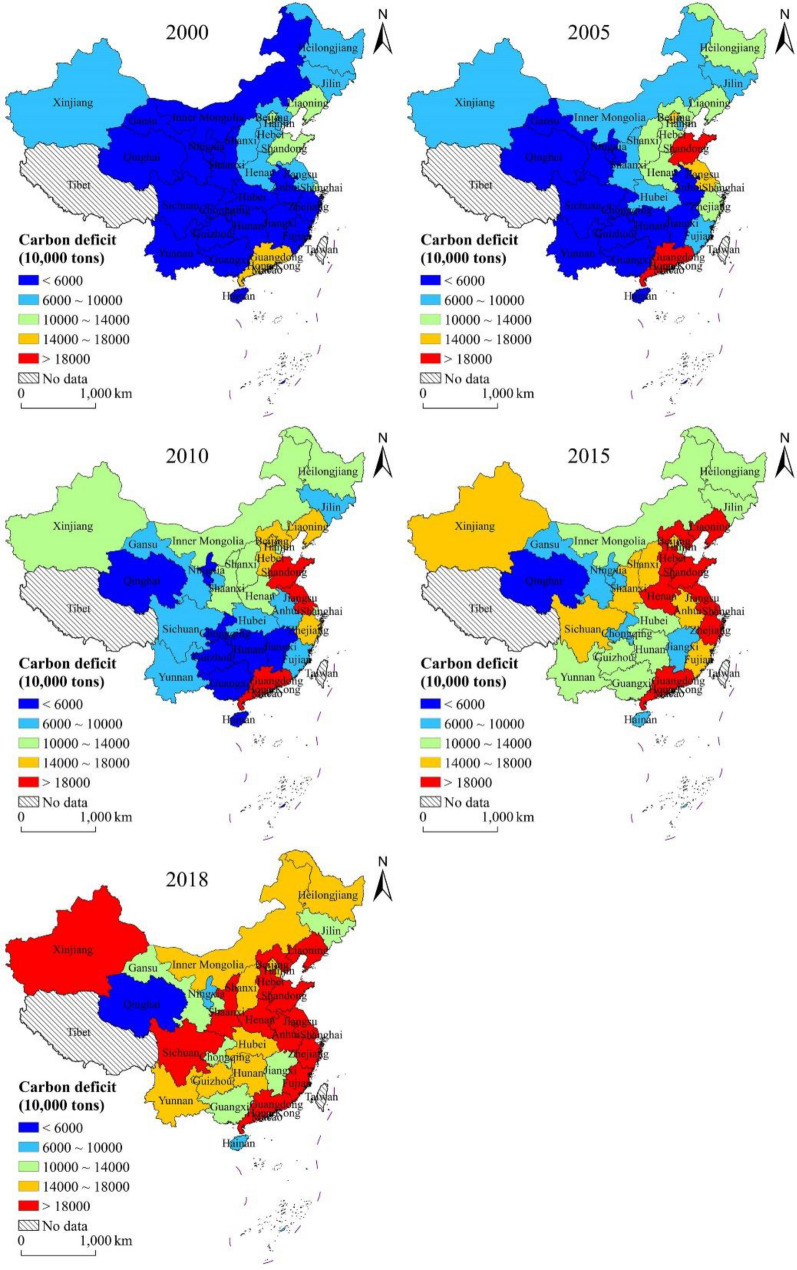
Spatial distribution of carbon deficit of energy consumption at provincial level

Based on estimations using the carbon emission model, carbon absorption model, and the carbon deficit model, the evolution of the spatial heterogeneity of carbon emissions, carbon absorption, and carbon deficit of energy consumption in the study area for 2000–2018 were obtained (Figs. 2, 3, 4). Due to variability in the level of economic development, population, energy consumption, and land use pattern in each provincial administrative region, there are obvious differences in the spatial distribution characteristics of carbon emissions, carbon absorption, and carbon deficit.

As can be seen in Fig. 2, the highest carbon emissions in 2000 were in Guangdong Province, followed by Beijing City, Shanghai City, Shandong Province, and Liaoning Province, while the carbon emissions of other provincial administrative regions were relatively low. The regions with the highest carbon emissions in 2005 were Guangdong Province and Shandong Province, followed by Beijing City and Jiangsu Province. The carbon emissions in eastern China in general increased significantly during 2005–2010. The change in the spatial distribution characteristics of carbon emissions also reflects the changing trend in the spatial distribution of China’s socio-economic development. By 2018, the regions with high carbon emissions further increased, with the carbon emissions of most provinces exceeding 1.40 × 10^7^ tons.

As can be seen in Fig. 3, the spatial distribution of carbon absorption in the study area remained stable from 2000 to 2018. The areas with the highest carbon absorption are the Sichuan, Yunnan, Guangxi, Heilongjiang, and Inner Mongolia Provinces; they have relatively larger administrative areas and a higher proportion of land use types that play a greater role in carbon absorption, leading to higher amounts of carbon absorption compared to other provincial administrative areas. The areas with low carbon absorption are the cities of Beijing, Tianjin, and the provinces of Shanghai, Shandong, Henan, Jiangsu, and Anhui—which are clustered together—and the discrete areas of Ningxia Hui Autonomous Region, Chongqing City and Hainan Province. The low carbon absorption is mainly due to two reasons: some provincial administrative regions have a small area and their contribution to carbon absorption is limited; the low carbon absorption in the rest of the provincial administrative regions is due to the fact that the proportion of land use types with strong carbon absorption capacity is relatively small in these areas, resulting in low total carbon absorption.

As shown in Fig. 4, the highest carbon deficit in 2000 occurred in Guangdong Province, followed by Beijing City, Shanghai City, Shandong Province, and Liaoning Province, while other provincial administrative regions had relatively low carbon deficit. In 2005, regions with the highest carbon deficit were Guangdong Province and Shandong Province, followed by Beijing City and Jiangsu Province, and the overall distribution pattern began to show high values in the north and low values in the south. By 2010, the areas included in the highest range of carbon deficit of more than 18 000 tons in Jiangsu Province increased compared to 2005, and the carbon deficit in the eastern region of China increased significantly. In 2015, the regions in the highest carbon deficit bracket increased to seven provincial administrative regions, mainly in the eastern part of China. In 2018, the number of regions with high carbon deficit increased further, spreading to the central and western regions on the basis of the strip-like clustering distribution in the east, and the number of regions with the highest carbon deficit increased to 13, with only Qinghai Province maintaining a carbon deficit of less than 60 million tons from 2000 to 2018.

### Spatial autocorrelation

To verify the existence of spatial autocorrelation of the carbon deficit in the study area at the provincial scale, the spatial Moran’s *I* was chosen. The global Moran’s *I* for the carbon deficit was calculated using ArcGIS 10.3 software (Environmental Systems Research Institute) to analyze the spatial autocorrelation of the carbon deficit in each provincial administrative region (Table 3).

**Table 3:** Results of global correlation analysis

	2000	2005	2010	2015	2018
*Moran’s I*	0.182	0.188	0.295	0.215	0.216
*z*	1.968	2.021	2.960	2.303	2.298
*p*	0.049	0.043	0.003	0.021	0.022

It can be seen from Table 3 that the index, global Moran’s *I*, for the provincial-scale carbon deficit in the study area was positive from 2000 to 2018. The global Moran’s *I* increased from 0.182 to 2000 to 0.295 in 2010, and then declined to 0.216 in 2018, with all *Z* values greater than 1.96, and all *p*-values significant at the 5% level, passing the significance test. This indicates that the carbon deficit at the provincial scale in the study area had a positive spatial correlation, and the degree of positive correlation first increased and then decreased.

**Fig. 5:**
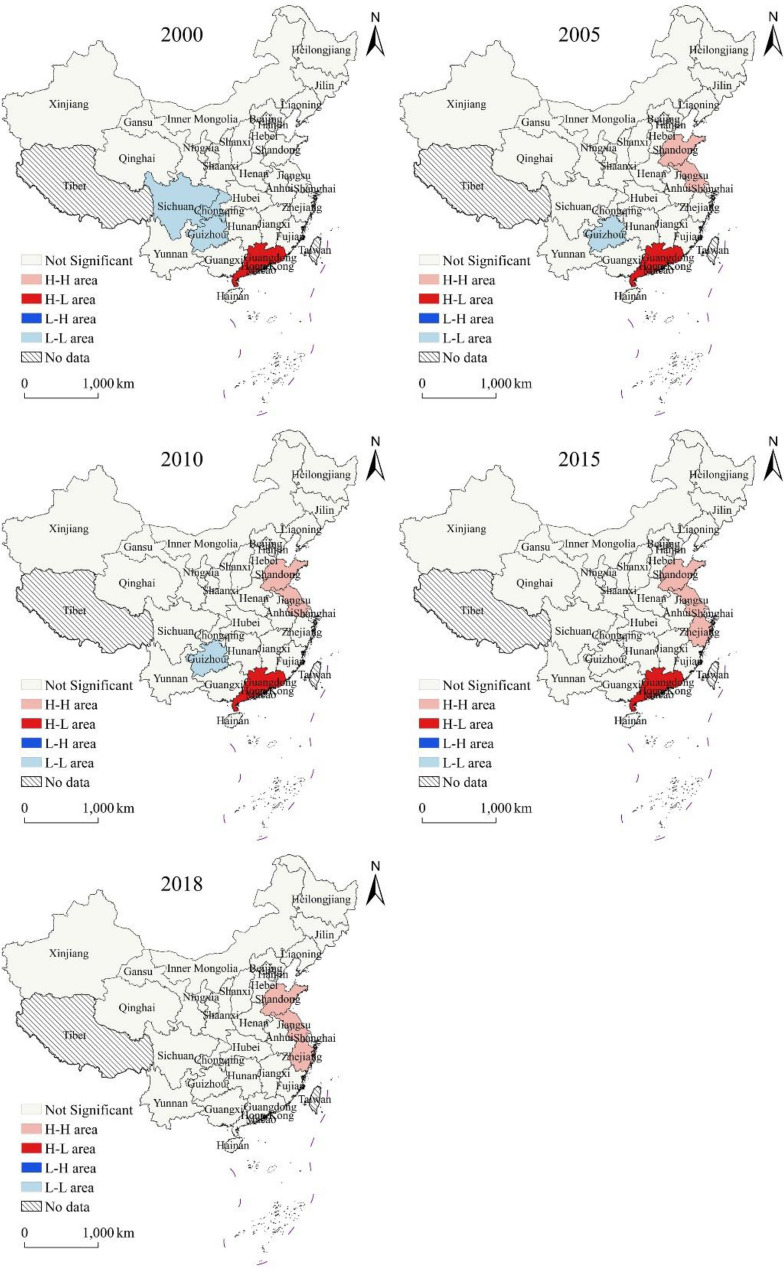
Local spatial correlation of carbon deficit of energy consumption at provincial level

LISA plots were used to analyze the local spatial autocorrelation of the carbon deficit (Fig. 5). In 2000, there were two types of agglomerations in the study area: the H-L area and L-L area. The L-L area included Sichuan Province, Guizhou Province, and Chongqing City, indicating that the overall carbon deficit in Southwest China was relatively low. Only Guangdong Province was an H-L area, and its carbon deficit was greater than that of neighboring provincial administrative regions. In 2005, only Guangdong Province was still an H-L area, while the L-L area included only the Guizhou Province; new H-H area was added, including Shandong Province and Jiangsu Province. The local spatial autocorrelation between 2010 and 2005 was consistent, indicating that the overall spatial distribution pattern of carbon deficit changed little from 2005 to 2010. In 2015, the H-L area remained unchanged, the L-L area disappeared, and the H-H area expanded further, adding Zhejiang Province to the H-H area for the first time, indicating that the overall carbon deficit had increased. In 2018, Guangdong Province changed from H-L area to not significant area, indicating that the carbon deficit in the provincial administrative regions around Guangdong Province grew rapidly, and there was only one type of spatial aggregation in the study area.

### Decoupling status

**Fig. 6:**
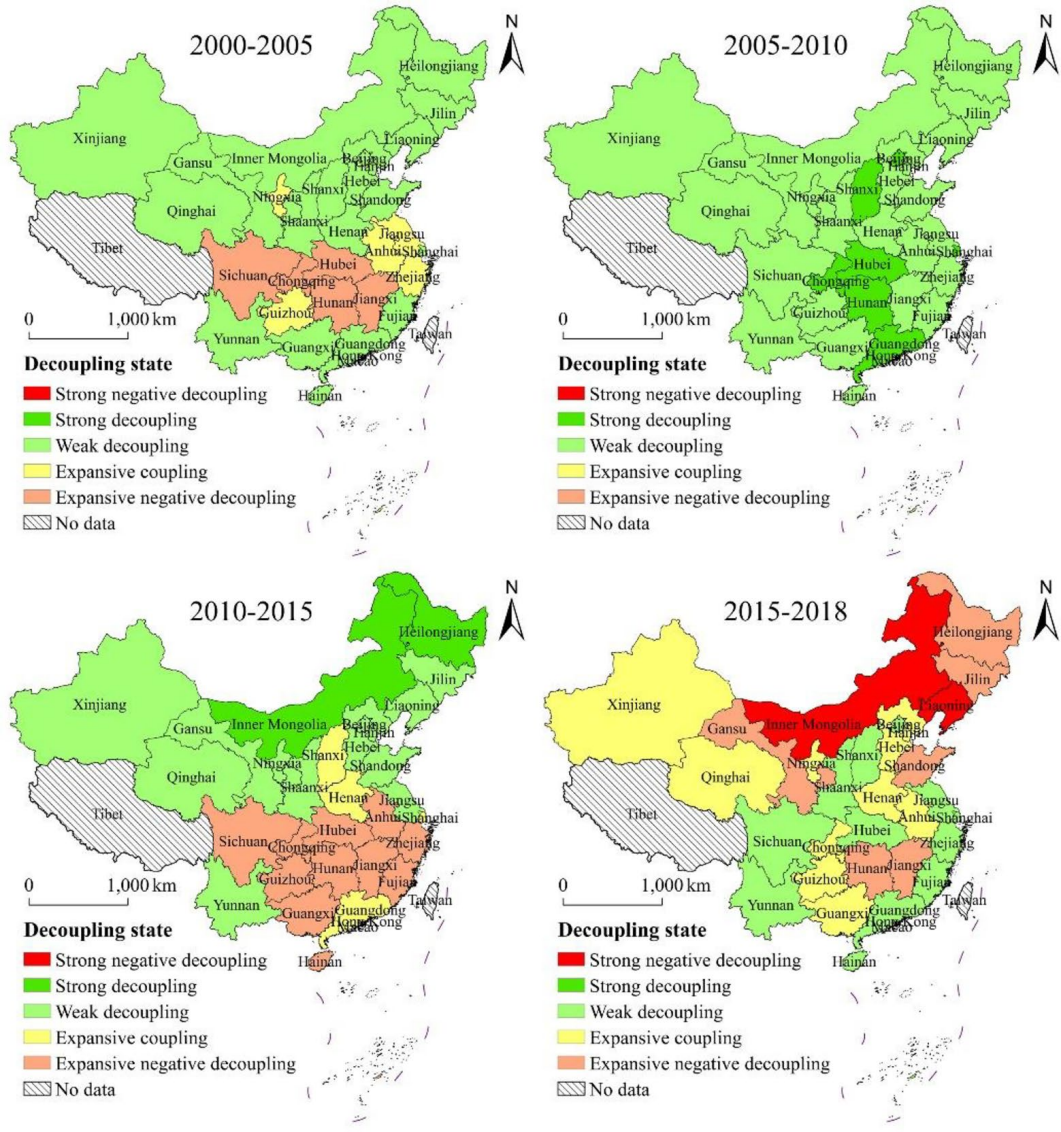
Spatial distribution of decoupling status at provincial level

Using the decoupling formula, the integrated decoupling index for carbon emissions and economic development, for provincial administrative regions within the study area for 2000–2018 was calculated, and the elasticity characteristics of carbon emissions in the study area were analyzed in conjunction with the criteria for classifying the decoupling into various types (Fig. 6). It should be noted that at no time was there a simultaneous decrease in the amount of carbon emissions and GDP, the decoupling status were therefore classified into five categories.

As can be seen in Fig. 6, the spatial distribution of the decoupling status of the study area showed significant differences over the four time periods. Overall, from 2000 to 2018, the decoupling status of provincial administrative regions in the study area was dominated by weak decoupling and expansive negative decoupling, with distribution range of weak decoupling area showing a decreasing trend. From 2000 to 2005, the study area was mainly dominated by weak decoupling, with 20 provincial administrative districts exhibiting weak decoupling, five showing expansive coupling with clustered distribution, and five indicating expansive negative decoupling. From 2005 to 2010, the study area was still mainly dominated by weak decoupling, and provincial administrative districts with weak decoupling further expanded—increasing to 23—while the remaining seven provincial administrative regions were in a state of strong decoupling. It can be seen that during this period, the overall carbon emissions of the study area indicated good decoupling from economic development. From 2010 to 2015, the decoupling status deteriorated compared to the previous period, and the study area was mainly dominated by weak decoupling and expansion of negative decoupling, in 14 and 10 provincial administrative regions, respectively. The expansion of negative decoupling was widely clustered and distributed in the southern China. From 2015 to 2018, the composition of the decoupling status of the study area changed significantly compared to that during 2010–2015, with 12, 10, six, and two provincial administrative regions exhibiting weak decoupling, expansive coupling, expansive negative decoupling, and strong negative decoupling, respectively. Moreover, the spatial distribution of the decoupling status also changed significantly; the southern region showed a clustered distribution of expansive negative decoupling during 2010–2015, and the situation in the region improved during 2015–2018, with most provincial administrative districts shifting to weak decoupling and expansive coupling. The northern part of the study area, on the other hand, displayed the opposite trend, showing weak and strong decoupling during 2010–2015, with a shift toward expansive coupling, expansive negative decoupling, and strong negative decoupling during 2015–2018 in the provincial administrative regions.

### Effectiveness of contribution of influencing factors to decoupling

**Fig. 7:**
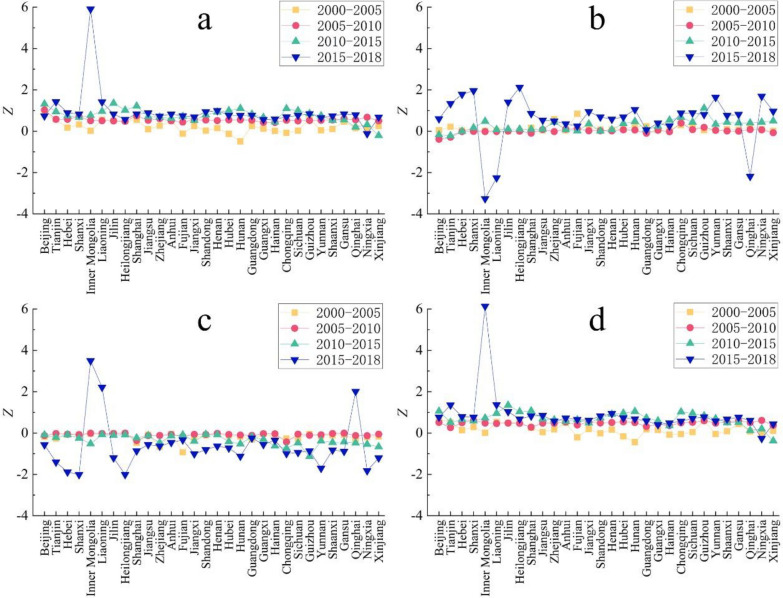
Effectiveness of contribution of influencing factors to decoupling. **a**: energy consumption intensity, **b**: population density of built-up land, **c**: area of built-up land, **d**: sum of the effectiveness of the three influencing factors

The degree of influence of energy consumption intensity, built-up land population density, built-up land area, as well as the three factors combined, on decoupling were measured by calculating the effectiveness of the contributions of influencing factors to decoupling, with the results shown in Fig. 7.

As shown in Fig. 7a, the effectiveness values of decoupling for energy consumption intensity, *Z*, in most provincial administrative regions were in the range of 0 to 1 during 2000–2018, indicating a weak contribution of energy consumption intensity to decoupling in most regions within the study area. From Fig. 7b it is seen that the effectiveness value corresponding to the population density of built-up land, *Z*, was also between 0 and 1 for this period, indicating a weak contribution to decoupling. With the evolution of time, the magnitude of the effectiveness value, *Z*, shifted from a relatively smooth to a fluctuating state, and the difference in effectiveness value, *Z*, among provincial administrative regions in the study area gradually became more pronounced. As shown in Fig. 7c, the trends in effectiveness values, *Z*, for built-up land area were opposite to those of energy consumption intensity and built-up land population density, with most provincial administrative regions having *Z* values between − 1 and 0. The differences in effectiveness values, *Z*, among provincial administrative regions in the study area also gradually became more pronounced with time. As can be seen from Fig. 7d, the total effectiveness value, *Z*, for the influencing factors for most provincial administrative regions were between 0 and 1, indicating that the individual contributions of the selected influencing factors to decoupling of carbon emissions from economic development in the study area were weak, but together, they still played a role in promoting decoupling.

### Scenario simulation analysis

Indicators are set for the three scenarios based on the trends in energy intensity, GDP per capita, population density, and built-up land area for the period 2000 − 2018 using the indicator setting method chosen for this study; the indicators are projected year by year based on Eq. (12). Details of the indicator settings for the three scenarios are presented in Table 4.

**Table 4:** Scenario indicator settings corresponding to annual average rate of change in contribution of each factor

	High-carbon scenario (%)	Basic scenario (%)	Low-carbon scenario (%)
*n*_*1*_(energy consumption intensity)	2.34	3.84	5.34
*n*_*2*_(GDP per capita)	7.29	5.79	4.29
*n*_*3*_(population density)	7.27	8.77	10.27
*n*_*4*_(built-up land area)	7.34	5.84	4.34

**Fig. 8:**
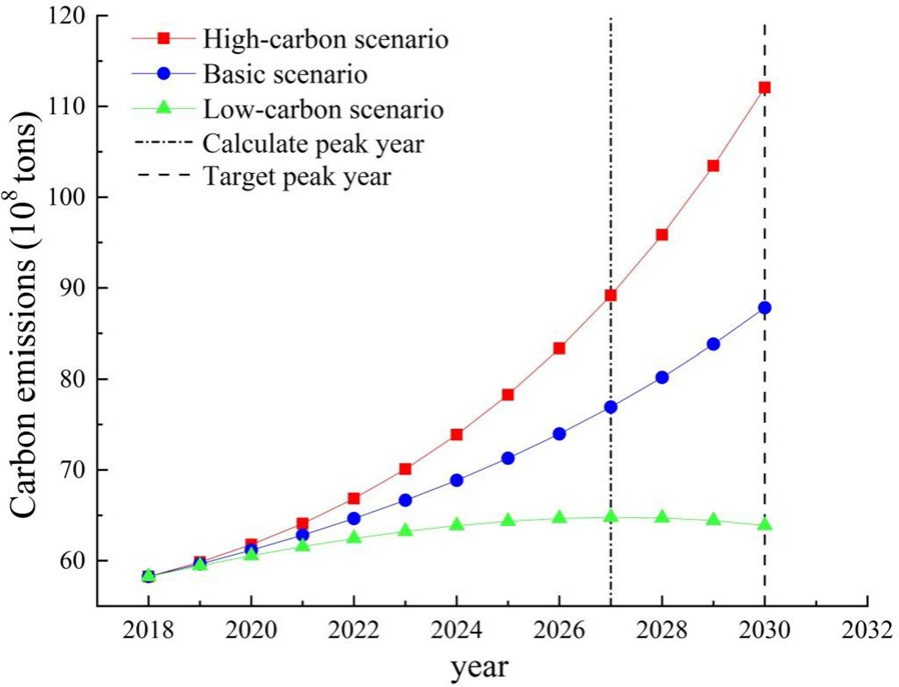
Carbon emissions prediction for study area under different scenarios

The future trend of carbon emissions in the study area for 2019–2030 is projected using the values of the specific indicators set as shown in Table 4, to predict the peak year of carbon emissions in the study area, and the results are shown in Fig. 8.

As shown in Fig. 8, there are significant differences among scenarios in the predicted carbon emissions in the study area. In particular, there is a carbon peak in the low-carbon scenario. The predicted peak year is 2027, which is three years earlier than the year expected by China for carbon emissions to peak, as part of its “dual carbon” goals. In the basic scenario and the high-carbon scenario, carbon emissions in the study area will continue to rise, and there will be no carbon peak phenomenon. In the high-carbon scenario, the push of the influencing factors toward increase in carbon emissions is enhanced and the inhibiting effect will be weakened; thus, carbon emissions will continue to increase significantly in the forecast period. In this scenario, carbon emissions in the study area are predicted to reach 11208.61 million tons by 2030, which is much higher than in the basic and low-carbon scenarios. In the basic scenario also, carbon emissions in the study area will show an upward trend for 2019–2030, but the upward trend is more moderate compared to that in the high-carbon scenario. In this scenario, the encouraging and inhibiting effects of none of the influencing factors on carbon emissions will be significant. Therefore, the growth rate of carbon emissions in the study area will be relatively low for the forecast period; carbon emissions in the study area are expected to reach 8781.78 million tons by 2030. In the low-carbon scenario, the boosting effect of the selected influencing factors on the increase in carbon emissions will be weakened, while the inhibiting effect will be enhanced; the rising trend curve of carbon emissions will be flat, and the carbon peaking phenomenon will appear in 2027. The peak carbon emissions in this scenario are 6479.27 million tons, which is 2439.06 million tons and 1213.91 million tons lower than the carbon emissions in the high-carbon scenario and basic scenario, respectively, for the same year.

## Discussion

Based on night light data and energy consumption statistics, this study calculated the combined carbon emissions from the energy consumption of 30 provincial administrative regions in China, to analyze the spatial heterogeneity and spatial aggregation distribution characteristics. In addition, the decoupling of carbon emissions from economic development was explored, and the effectiveness of different indicator factors on decoupling was quantified and analyzed. Finally, changing future trends of carbon emissions from energy consumption in the study area were predicted. Compared with previous studies, we have calculated the total carbon emissions of energy consumption and the carbon absorption of various land uses, and have an intuitive understanding of China’s overall carbon balance. The future carbon peak year is predicted according to scenarios, which has a feedback effect on the choice of carbon emission reduction path.

The estimation of carbon emissions using night light data can avoid errors caused by differences in the statistical robustness of the yearbook data, and can also reflect the socio-economic development of different regions [56]. By fitting energy consumption statistics with night light data, the influence of socio-economic factors on carbon emissions was incorporated in the estimation. The trend of carbon emissions as revealed by the data is relatively more in line with reality [57]. In addition, although there may be some degree of error in fitting night light data, it is within an acceptable range. On this basis, further analysis of spatiotemporal changes at the city, county, and grid scales can be conducted in subsequent research, which has more potential for in-depth research compared to statistical data [58, 59]. In terms of spatial heterogeneity, carbon emissions in eastern China increased rapidly during the study period. The study area gradually showed a distribution pattern with high emissions in the east and low emissions in the west. Guangdong Province was one of the regions with the highest emissions, while a unipolar pattern prevailed in southern China, which is similar to the results of previous studies [60, 61]. This is mainly due to the large population density and high level of industrial development in the eastern region and Guangdong Province, which leads to large energy consumption and relatively high carbon emissions [62]. In addition, China’s provincial-scale carbon emissions showed positive spatial autocorrelation during the study period, which is also consistent with the results from previous studies [63]. In the decomposition of the influencing factors, the rapid economic development and expansion of built-up land had an encouraging effect on the increase in carbon emissions in China. At present, China’s economic development still needs to be driven by various factors and requires large-scale energy consumption, and built-up land is the main carrier of economic activities [64, 65]. Therefore, economic development and expansion of built-up land can lead to a substantial increase in carbon emissions [66]. On the other hand, the reduction of energy consumption intensity and the decrease in population density in built-up land played an important role in reducing carbon emissions. The reduction of energy consumption intensity means the transformation of China’s economic development mode and the improvement of energy technology. Since the main source of carbon emissions is energy activities, reducing energy consumption intensity means reducing the corresponding carbon emissions [67]. The increase in population density in built-up land will directly increase energy consumption, thus increasing carbon emissions. On the contrary, its decline can promote the reduction of carbon emissions [68]. These results are consistent with previous studies [69, 70]. China’s overall economic development is stable and rapid, and the urbanization and industrialization processes in China are not yet complete [71]. The economic development of the study area will remain in a state of continuous and stable growth in the future, and the expansion of built-up land will continue. Therefore, it is necessary to optimize the economic structure, transforming the economic development mode, and formulating policies to control the expansion of built-up land to the maximum extent considering regional differences [72]. Energy as a basic element of growth supports the rapid development of the economy, which in turn leads to a larger consumption of energy and an increase in carbon emissions. Therefore, the increase in total carbon emissions is concomitant with economic development [73]. Efforts must be made to improve technology and promote industrial restructuring, to effectively reduce energy consumption intensity [74, 75]. High-carbon emission areas are mainly distributed in the eastern region. This is mainly because the industrial structure of these provinces is dominated by the secondary industry. Therefore, the eastern region should focus on optimizing the industrial structure, developing technology-intensive industries and replacing high-emission industries. For the western region of China, it may undertake the energy-intensive industries in the eastern region, which are potential areas for carbon emission growth. Therefore, improving energy utilization efficiency is effective for carbon emission reduction in the region. It is important for China to achieve the “dual carbon” targets by predicting future changes in carbon emissions. The peak year of carbon emissions from energy consumption, as predicted in this study, is 2027, which is similar to the peak year obtained in previous studies, and is within a reasonable time range to achieve China’s target [76].

## Conclusions

An in-depth study on the spatial heterogeneity of the overall carbon budget of energy consumption and its influencing factors and the status of decoupling carbon emissions from economic development, and undertaking simulation predictions by setting up different scenarios is crucial for China to achieve the targets of carbon emissions peaking by 2030 and carbon neutrality by 2060. This study found that both carbon emissions and carbon deficit showed a continuous upward trend, and the spatial distribution characteristic changed to high in the east and low in the west, while the spatial distribution pattern of carbon absorption remained stable. The carbon deficit of energy consumption had a significant positive spatial correlation; the degree of positive correlation first strengthened and then weakened, but the distribution range of spatial aggregation was small. The study area as a whole was dominated by weak decoupling and expansion negative decoupling with a decreasing trend. Overall, energy consumption intensity, built-up land area, and population density in the built-up land area influenced the decoupling between carbon emissions in energy consumption and economic development. Only in the low-carbon scenario will the study area achieve the peak carbon emissions target ahead of schedule, in 2027, with the peak carbon emissions at 6479.27 million tons.

This study has its shortcomings: while most of the region display a good fit between energy consumption statistics and night light data, the fitting results of carbon emissions for a few socio-economic developed areas were much higher than the actual value. It is necessary to further improve the simulation method to make the model fitting more accurate. On the other hand, the carbon emissions investigated in this study were only due to energy consumption, while carbon emissions from other activities, such as land use and industrial production processes, are also important sources of total regional carbon emissions. In subsequent studies, other sources of carbon emissions must be included in the estimation model to more accurately simulate and predict the evolution of overall regional carbon emissions. In future, we plan to continue to improve the model estimation methods, and include more sources of carbon emission to assess the spatial and temporal evolution of the carbon budget in the study area more comprehensively and accurately.

## Data Availability

Night light data for the study area for 2000, 2005, 2010, 2015, and 2018 were downloaded via the Harvard Dataverse platform (10.7910/DVN/YGIVCD) with a resolution of 500 m × 500 m, and subsequently pre-processed by re projection and cropping to obtain night light data with a resolution of 1 km × 1 km for the study area. The data pertaining to various land use types were downloaded from the Resource and Environment Science and Data Center (http://www.resdc.cn/Default.aspx); the Chinese land use remote sensing monitoring data for 2000, 2005, 2010, 2015, and 2018 were selected, which are 1 km × 1 km raster data generated by manual visual interpretation using the Landsat TM/ETM remote sensing images. According to the land use classification table provided by the data source website, it is reclassified into six land use types, namely cultivated land, forestland, grassland, water land, built-up land, and unused land. According to the attributes, forestland, grassland, water land, and unused land are extracted for carbon absorption calculation. The socio-economic data required for the calculation of energy consumption and other related parameters, as well as data on relevant indicators affecting carbon emissions were obtained from “China Statistical Yearbook,” “China Energy Statistical Yearbook,” and relevant provincial and municipal statistical yearbooks for 2001, 2006, 2011, 2016, and 2019.

## Abbreviations

DEA: Data envelopment analysis
LMDI: Logarithmic mean divisia index
DN: Digital number
H-H: High-observed area surrounded by high-observed areas
L-L: Low-observed area surrounded by low-observed areas
L-H: Low-observed area surrounded by high-observed areas
H-L: High-observed area surrounded by low-observed areas
